# Long Noncoding RNA Expression Rofiles Elucidate the Potential Roles of lncRNA- XR_003496198 in Duck Hepatitis A Virus Type 1 Infection

**DOI:** 10.3389/fcimb.2022.858537

**Published:** 2022-04-22

**Authors:** Nana Sui, Ruihua Zhang, Yue Jiang, Honglei Yu, Guige Xu, Jingyu Wang, Yanli Zhu, Zhijing Xie, Jiaqing Hu, Shijin Jiang

**Affiliations:** ^1^ College of Veterinary Medicine, Shandong Agricultural University, Taian, China; ^2^ Shandong Provincial Key Laboratory of Animal Biotechnology and Disease Control and Prevention, Taian, China; ^3^ Shandong GreenBlue Biotechnology Co. Ltd., Taian, China

**Keywords:** long non-coding RNA, cis-target, trans-target, network, innate immune response, autophagy

## Abstract

Duck hepatitis A virus type 1 (DHAV-1) is a highly lethal virus that severely affects the duck industry worldwide. Long noncoding RNAs (lncRNAs) exert crucial roles in pathogen attacks. Here, we conducted deep transcriptome analysis to investigate the dynamic changes of host lncRNAs profiles in DHAV-1-infected duck embryo fibroblasts. We identified 16,589 lncRNAs in total and characterized their genomic features. Moreover, 772 and 616 differentially expressed lncRNAs (DELs) were screened at 12 and 24 h post-infection. Additionally, we predicted the DELs’ cis- and trans-target genes and constructed lncRNA-target genes regulatory networks. Functional annotation analyses indicated that the putative target genes of DELs participated in diverse vital biological processed, including immune responses, cellular metabolism, and autophagy. For example, we confirmed the dysregulation of pattern recognition receptors (TLR3, RIG-I, MDA5, LGP2, cGAS), signal transducers (STAT1), transcription factors (IRF7), immune response mediators (IL6, IL10, TRIM25, TRIM35, TRIM60, IFITM1, IFITM3, IFITM5), and autophagy-related genes (ULK1, ULK2, EIF4EBP2) using RT-qPCR. Finally, we confirmed that one DHAV-1 induced lncRNA-XR_003496198 is likely to inhibit DHAV-1 replication in DEFs. Our study comprehensively analyzed the lncRNA profiles upon DHAV-1 infection and screened the target genes involved in the innate immune response and autophagy signaling pathway, thereby revealing the essential roles of duck lncRNAs and broadening our understanding of host-virus interactions.

## Introduction

Duck virus hepatitis (DVH) is a highly lethal and fast-spreading disease affecting ducklings, which is caused by the duck hepatitis A virus (DHAV) (Chalmers and Woolcock, 1984; Yang et al., 2012; Zhang et al., 2020a). As the only *Avihepatovirus* genus in the Picornaviridae family, the DHAV genus is genetically classified into three types: DHAV-1, DHAV-2, and DHAV-3 (Tseng et al., 2007; Gao et al., 2012). Among the causative agents of DVH, DHAV-1 is the most common and widely distributed and is responsible for acute hepatitis characterized by neurological symptoms and ecchymotic hemorrhages on liver surfaces (Song et al., 2014; Ou et al., 2017). In detail, DHAV-1 transmission occurs in the digestive and respiratory tracts *via* breath and direct contact between ducklings with a morbidity rate of up to 90% (Ding and Zhang, 2007; Jin et al., 2008; Wen et al., 2018). Currently, DHAV-1 has become one of the major concerns in the duck industry (Fu et al., 2008; Zhang et al., 2020b).

High throughput sequencing allows us to accurately detect and identify both protein-coding and noncoding RNAs (ncRNAs) (Stark et al., 2019). Advanced genome- and transcriptome-wide analysis indicated that only 1%–2% of the genome encodes proteins, while over 75% is actively transcribed into non-protein-coding RNAs (Carninci et al., 2005). According to the sequence length cutoff, ncRNAs are classified into two subclasses: small ncRNA (sncRNA, < 200 nucleotides) and long ncRNAs (lncRNAs, > 200 nucleotides). These ncRNAs exert central modulatory roles in gene expression regulation, RNA editing, and epigenetic activities (Hombach and Kretz, 2016). To date, transcriptome sequencing combined with bioinformatics analysis has identified many new mRNAs and ncRNAs in animals coping with multiple pathogen attacks (Hu et al., 2016; Agliano et al., 2019), which could help uncover novel pathogenic mechanisms of host-pathogen interactions.

Based on their genome location and direction relative to nearby protein-coding genes, lncRNAs can be designated as five types: sense, antisense, intergenic, intronic, and bidirectional (Iyer et al., 2015). LncRNAs frequently act as molecular signals, scaffolds, decoys, or competing endogenous RNAs to affect gene expression and perform regulatory roles (Chen et al., 2017; Yao et al., 2019). Accumulating evidence suggests that lncRNAs serve as versatile regulators during diverse biological and physiological processes, especially in host-pathogen interactions against various infectious agents (Agliano et al., 2019). For example, lnc-THRIL is a nuclear lncRNA that forms complex with heterogeneous nuclear ribonucleoprotein L (hnRNPL) to trans-regulate tumor necrosis factor α expression by binding to its promoter (Li et al., 2014). Long intergenic ncRNA (lincRNA)-COX2 is another trans-acting lncRNA that regulates both the activation and repression of the inflammatory gene cyclooxygenase 2 by interacting with hnRNP-A/B and hnRNP-A2/B1 (Carpenter et al., 2013). Meanwhile, lnc-LUCAT1 inhibits type I interferon (IFN-I) production by interacting with STAT1 in the nucleus, thereby suppressing the antiviral immune response (Agarwal et al., 2020). Recent reports have emphasized the importance of lncRNAs, particularly in modulating host-pathogen interactions (Agliano et al., 2019; Hao et al., 2021). However, limited information on lncRNAs’ expression and function in ducks during DHAV-1 attack.

In this research, we first analyzed the lncRNA expression profiles in DHAV-1-infected duck embryo fibroblasts (DEFs), explored the lncRNAs’ genomic structure, and screened the differentially expressed lncRNAs (DELs) transcripts for target genes predicting. Additionally, DELs and trans-target genes were obtained to establish co-expression networks. Gene Ontology items (GO) and Kyoto Encyclopedia of Genes and Genomes (KEGG) analysis revealed that many target genes participated in immune-related and autophagy-related pathways. Finally, we demonstrated the duck lncRNA-XR_003496198 (lnc-XR_003496198) as an antiviral factor against DHAV-1. Thus, our findings could help uncover the detailed pathogenesis of the DHAV-1 attack.

## Materials and Methods

### Cell Culture and DHAV-1 Infection

Fresh DEFs were isolated from 10-day-old SPF duck embryos and cultured as described previously (Lan et al., 2019). When cells reached 80%-90% confluence, the DEFs were stimulated with pathogenic DHAV-1 LY0801 strain (3 multiplicity of infection (MOI)). At 2 h post-infection (hpi), the inoculum was discarded and replaced with a fresh maintenance medium containing 2% chicken serum (CS, Solarbio, Beijing, China) and 1% penicillin and streptomycin. The cell lysates were harvested in triplicates at 12, 24, and 36 hpi to detect the viral load.

All animal experiments were conducted following the guidelines issued by the Animal Care and Use Committee of Shandong Agricultural University (Approval Number: # SDAUA-2018-045).

### RNA Extraction and Sequencing

DEFs’ total RNA was isolated following the standard protocol using the Trizol^®^ reagent (Ambion). RNA quantification and quality were evaluated using a Bioanalyzer 2100 system (Agilent Technologies, CA, USA). Stranded mRNA sample preparation and next-generation sequencing were conducted by Novogene (Beijing, PR China). A total of nine libraries (which contained three biological replicates) were constructed and labeled as N1, N2, N3, D1, D2, D3, H1, H2, and H3 (N: non-infected DEFs harvest at 0 hpi, D: infected DEFs harvested at 12 hpi, H: infected DEFs harvested at 24 hpi). Transcriptome sequencing was performed on an Illumina Hiseq 2500 platform and PE150 (paired-end) runs. Clean reads were obtained according to the manufacturer’s instruction and were then mapped to the genome of Anas platyrhychos using HISAT2 (Kim et al., 2015). Finally, the aligned reads were transferred to StringTie (Pertea et al., 2016) for transcript assembly.

### Identification of DEFs LncRNA

The resulting transcripts were merged using Cuffmerge (Ghosh and Chan, 2016) and then identified lncRNAs from the assembled transcripts. Only transcripts with length > 200 nt and exon > 2 were retained, and the transcripts were then matched with a reference annotation using Cuffcompare (Ghosh and Chan, 2016). The Coding potential was predicted using CNCI (Sun et al., 2013), CPC (Kong et al., 2007), and Pfam-scan (Finn et al., 2014; El-Gebali et al., 2019). The transcripts that failed to encode any proteins were considered as candidate lncRNAs. Moreover, class code clustered candidates lncRNAs into lincRNA, intronic lncRNA, and antisense lncRNA. The transcripts’ genomic features (length, exon length, and exon number) between different lncRNA types were compared.

### Differential Expression Analysis

The DESeq R package analyzed DELs of the control and DHAV-1 infection groups. The mapped outputs were counted using StringTie software, and expression values were normalized using Reads Per kilobase of transcript per Million mapped reads (RPKM) (Pertea et al., 2016). DELs with | log2 (Fold Change) | > 0 & padj < 0.05 were filtered for further target genes prediction.

### lncRNA-Target Genes Prediction and Functional Annotation

The DELs’ target genes were predicted by applying two algorithms: the cis- (co-location) and trans-acting (co-expression) modes. The protein-coding genes near the position of lncRNA within 10/100kb were considered potential cis- targets.

Functional annotation enrichment analyses (GO and KEGG) for the cis- and trans- target genes were implemented using the cluster Profiler R package. The Go terms and KEGG pathways were considered significant when the corrected *P*-value was < 0.05.

### Quantitative Real-Time PCR

Total RNA was isolated by the method mentioned above, cDNA was then generated using a reverse transcription kit (UE, China), and RT-qPCR was performed on a Light Cycler 480II instrument (Roche, Basel, Switzerland) using SYBER Green Real-Time PCR Kit (TaKaRa, Dalian, China) following the manufacturer’s protocols.

Primers were either synthesized based on the reference sequences using NCBI primer BLAST or from published literature (**Table 1**). The DHAV-1 virus copies were identified following the method established by our laboratory (Lin et al., 2016). The data were normalized *via* the comparative 2^-ΔΔCT^ method using endogenous β-actin (ΔCt) as a control (Livak and Schmittgen, 2001).

**Table 1:** Primers used in this study.

Primer	Forward (5′-3′)	Reverse (5′-3′)	Resource
STAT1	GTAAAGAGGGAGCAATCA	TATCAGGGAAAGTAACAGC	(Qian et al., 2018)
IL6	TTCGACGAGGAGAAATGCTT	CCTTATCGTCGTTGCCAGAT	(Song et al., 2014)
RIG-I	GCTACCGCCGCTACATCGAG	TGCCAGTCCTGTGTAACCTG	(Li et al., 2015)
MDA5	CCTGGAGTAGGAGGTGCAAA	CAGTTGGGAGGCATGTTCTT	(Song et al., 2014)
TLR3	CCTGGATTGAGGTCCCAGTA	TTCTCCACCCTTCAAAATGG	(Song et al., 2014)
IFITM1	CACCGCCAAGTACCTGAACA	CGATCAGGGCGATGATGAG	(Song et al., 2014)
TRIM25	GCCTCAGCTCCGCAAGAACAC	ACCGCCTCATCTTCCTCATCCTC	XM_005012325
IRF7	CGCCACCCGCCTGAAGAAGT	CTGCCCGAAGCAGAGGAAGAT	(Chen et al., 2019)
STING	ACAAGCACAGCCTCTACGCAATC	CGCAATGAGCCTGTAGGTTCC	(Cheng et al., 2019)
LGP2	AAGTTCATCCACCCTAAATC	GCATCAGAATTGAGCTGAGC	XM_038168878.1
IL10	CAACCTGCTGCTGAGCCTGAAG	CGCCTTGTAGATGCCGTTCTCG	NM_001310368
IFITM3	AGGACAGCCAGGAGCCTCAAC	ACTGCCAGAATGACCACAAGGATG	KF584228
IFITM5	TGGTGCTGCCTCTCCTGCTC	TGTCCTCATCGTCGCTTGTGTTG	NM_001310780
TRIM35	TCAACCACACGCTCAGCAACC	TTGTCGTCCAGGCAGAAGAGTTTG	XM_027455469
TRIM66	CTGCTCCAGTTGGTGATGCTCTC	TCTTGCGAGTGCCGTTGCTAAG	XM_038180488
ULK1	TCAACAGCATCACAGCAGAGAAGC	CTTCCATCAGCAGCAGAGCCTTG	XM_038187281
ULK2	AACTTGCTGGCTCTGGCTAATCG	TGAAATCCCTTGGTTCGGTGATGTC	XM_027472676
EIF4EBP2	CAGCAGCCTGAGCAATCACGAC	AAATCTGGGAAATGGCGAAGGAGAG	XM_027460510
β-actin	CACAGATCATGTTTGAGACCTT	CATCACAATACCAGTGGTACG	(Xie et al., 2019)
lnc-TCONS_00037101	GATGGCAGCAGGCAGTTCAGAG	TGATGGCAGAGCAGGGAGGTTC	Designed by ourself
lnc-XR_001194122	GCCTTGGTGATTGTGCAACG	GGTTGCCTCATGGCAGCTTT	Designed by ourself
lnc-XR_001193397	CCACCAGAGCAAGCAGCAAGAG	AGAGGCAGAGGATTGGCAGAGG	Designed by ourself
lnc-TCONS_00113417	GCACGCTTCAGGATCTCCACAG	TCTGAGCAAAGAACCCAACAACCC	Designed by ourself
lnc-XR_003501236	ACTGCTGATGCCTCTGCGAAAC	ATTACACACCCACACCCACAAGTC	Designed by ourself
lnc-XR_003496198	TGGCTGAGTGTTAGAAGGGTAGGAC	GTCACTCCAACTCCAGCATAACTCC	Designed by ourself
Lnc-XR_002405515	CCAGTCCTGAGCAAAGAGCAACC	GGGAGGCTGGGTGAAACAAAGAG	Designed by ourself

### Plasmids Construction and Transfection

The selected lncRNAs were amplified from DEFs by traditional PCR, and the PCR products were subcloned into the PCAGGS-flag plasmid with a homologous recombination kit (New Cell & Molecular Biotech Co, Ltd, China) and sequenced by Sangon (Shanghai, China). The small interference RNA (siRNA) targeting lnc-XR_003496198 and negative control siRNA were obtained from Genepharma (shanghai, China). Knockdown efficiency of the target lncRNAs was verified by RT-qPCR analysis.

DEFs seeded in six-well plates were transfected with the abovementioned plasmids, then stimulated with DHAV-1 for 24 h. The cell lysates were used for immunoblotting, and the supernatant was prepared for viral titers detection.

### Viral Replication Detection and Western Blot

DEFs grown in 96-well plates were used for viral titration in triplicate with 10-fold serial dilutions. The virus titers were determined by quantifying TCID50 and calculated using Reed and Muench method. DEFs were suspended in RIPA buffer (Beyotime, China), including protease inhibitor cocktail, to investigate DHAV-1 expression at the protein level. The cell mixtures were loaded onto 12.5% sodium dodecyl sulfate-polyacrylamide gel electrophoresis and were then electroblotted to the nitrocellulose membrane. The membranes were blocked with 1% BSA followed by overnight incubation with primary antibodies (1:1000 dilutions) at 4°C. Finally, the proteins were visualized with an ECL detection kit (NCM, biotech) after incubating with HRP-conjugated anti-mouse IgG (1:1000 dilutions; Engibody, China).

### Statistical Analysis

Data were presented as means ± SD. Significant differences were performed using Student’s t-tests. Data analyses were evaluated using SAS version 9.0 (Cary, NC) and GraphPad Prism Software version 8.0 (San Diego, USA). * indicated *P* < 0.05 and ** indicated *P* < 0.01.

## Results

### Confirmation of DHAV-1 Infection in DEFs

**Figure 1:**
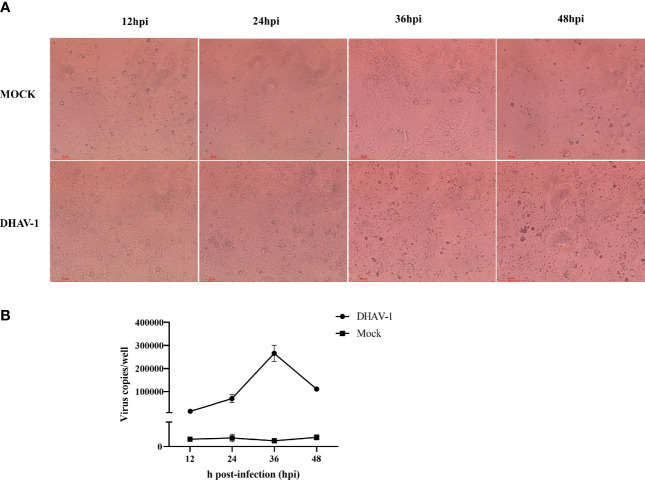
Characteristics of DEFs following DHAV-1 infection at 12, 24, 36, and 48 hpi. **(A)**. Represent morphology of mock-infected and DHAV-1-infected DEFs at 12, 24, 36, and 48 hpi. **(B)**. The viral copy numbers of DHAV-1 on DEF cells with an MOI of 3 at 12, 24, 36, and 48 hpi.

FCS (fetal calf serum) nonspecifically inhibits DHAV-1, whereas CS exerted no or minimal inhibitory influence (Chalmers and Woolcock, 1984). Therefore, according to a previous report, we tried to culture DHAV-1 in a maintenance medium with 2% CS (Wang et al., 2020). To identify DHAV-1’s replication efficiency in DEFs, we established the growth kinetics curve of the virulent LY0801 strain. The infection was monitored by observing cytopathic effects and virus copies. Compared to mock-infected cells without no cytopathic signs, the cytopathic effect (CPE) in infected DEFs started within 12 hpi, became more visible at 24 hpi, and the cells were crumbling and falling at 36 hpi (**Figure 1A**). DHAV-1 copies gradually increased before 36 hpi and decreased at 48 hpi (**Figure 1B**). Due to the severe shedding of infected DEFs at 36 hpi, we collected the DEFs at 12 and 24 hpi for transcriptome sequencing.

### RNA-Sequencing Overview

**Figure 2:**
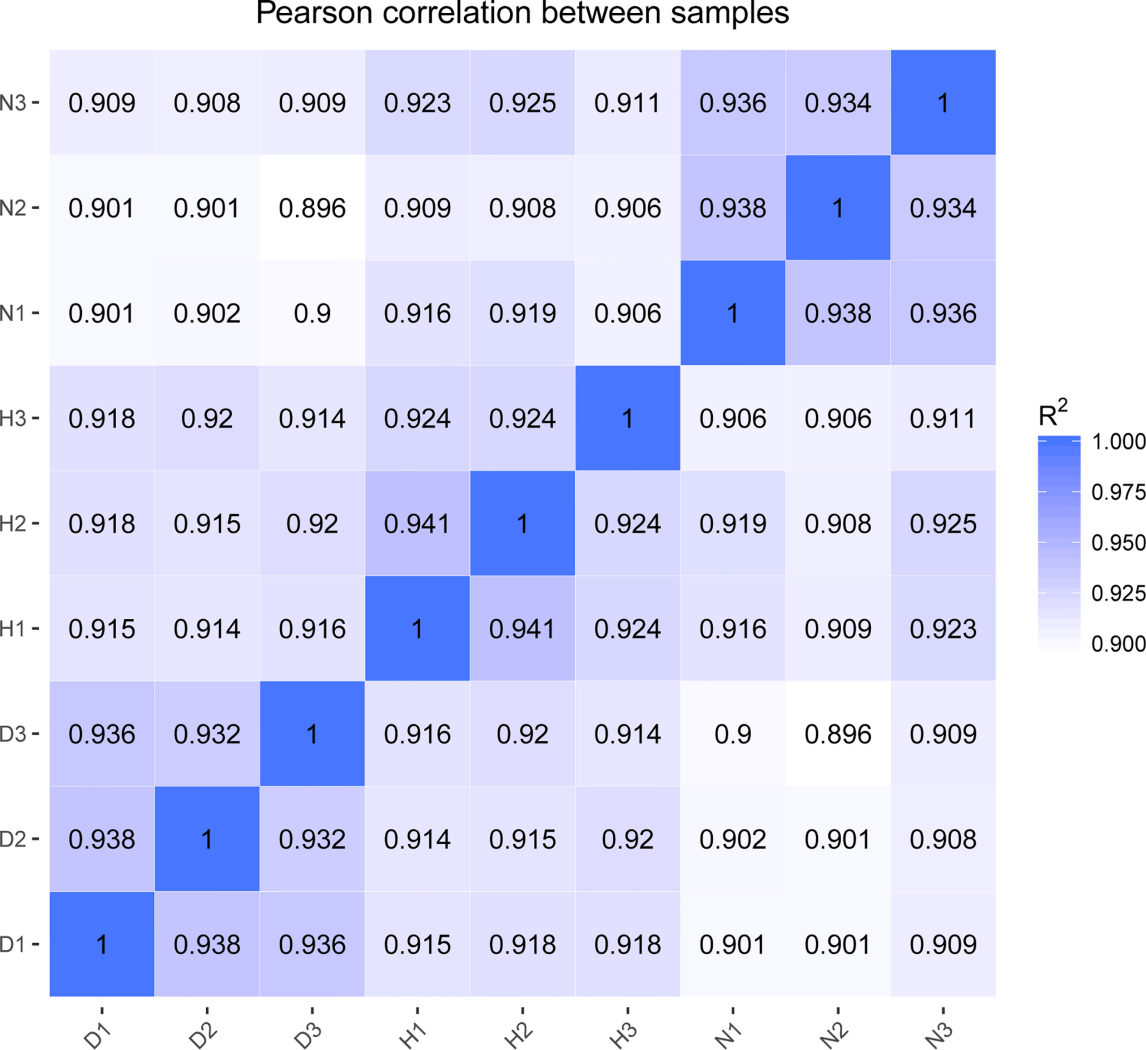
Heatmap shows Pearson’s correlation of expression levels between samples.

The Illumina Hiseq Xten platform was applied to profile lncRNA expression during DHAV-1 infection. More than 78 million raw reads were produced from each library (Accession number PRJNA715039). After initial processing, the clean reads comprised > 94% of the raw data, indicating the sequencing data’s high accuracy. Subsequently, the clean reads mapped to the reference genome were highlighted, demonstrating a high fraction of reading alignment accuracy (average alignment rate ≥ 86.49%) (**Table 2**). Moreover, Pearson’s correlation coefficients of all pairwise comparisons were > 0.90, attesting to the high consistency of the data (**Figure 2**). Taken together, the sequencing data and biological replicates indicated sufficient data quality for further analysis.

**Table 2:** Major characteristics of lncRNA libraries and reads mapping to the duck reference genome.

Sample name	Clean reads	Raw_bases (G)	Clean_bases (G)	Error rate (%)	Q20 (%)	Q30 (%)	GC content (%)	Total mapped
D1	83146498	12.67	12.47	0.02	98.3	94.99	52.72	71192894 (85.62%)
D2	92652030	14.2	13.9	0.02	98.09	94.47	54.97	79715666 (86.04%)
D3	82372074	12.57	12.36	0.02	98.22	94.81	50.47	71696338 (87.04%)
H1	80578846	12.24	12.09	0.02	98.22	94.84	50.66	68896911 (85.5%)
H2	88071804	13.37	13.21	0.02	98.24	94.85	50.49	75390420 (85.6%)
H3	78200424	11.89	11.73	0.02	98.15	94.73	52.67	65247232 (83.44%)
N1	78107302	11.92	11.72	0.03	97.81	93.81	52.65	69087865 (88.45%)
N2	84030114	12.85	12.6	0.02	98.02	94.43	52.97	73442464 (87.4%)
N3	89673650	13.65	13.45	0.03	97.99	94.02	51.59	79862798 (89.06%)

### Duck lncRNAs Characterization

**Figure 3:**
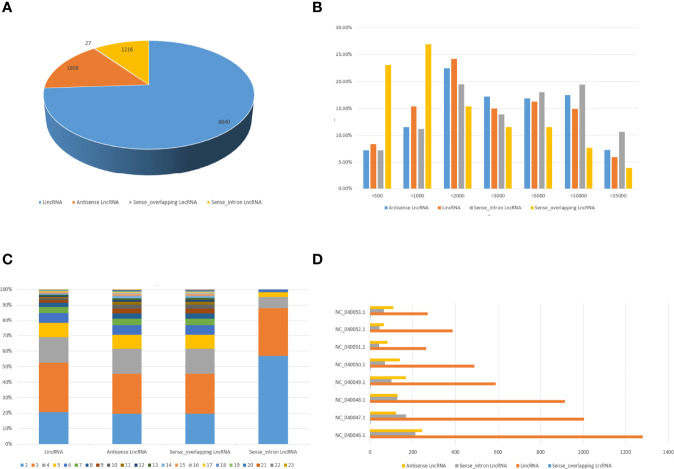
Characteristics of duck lncRNAs. **(A)** The number of lincRNAs, intronic lncRNAs, sense overlapping lncRNA, and antisense lncRNAs in DEFs. **(B)** Transcript length distribution of different categories of lncRNAs. **(C)** Analysis of exons numbers for different categories of LncRNAs. **(D)** Distribution of different categories of lncRNAs on different chromosomes.

To analyze the features of lncRNAs, we investigated the characteristics and transcription patterns based on the lncRNA profiles. A total of 11941 annotated lncRNAs and 4648 novel lncRNAs were identified. All detected lncRNAs were classified into four types, including 8840 intergenic, 1216 sense-intron, 1858 antisense, and 27 sense-overlapping lncRNAs (**Figure 3A**). Nearly half of duck lncRNAs were medium-length lncRNA (1000 – 5000 bp: 49.8%), 31.4% were short lncRNA (200 – 1000 bp), and only 18.1% were long lncRNAs (> 5 Kb) (**Figure 3B**). Moreover, the exons revealed that most lncRNAs had fewer than 5 exons (**Figure 3C**). The mapping of the lncRNAs to the duck chromosomes showed that all lncRNAs were distributed in each chromosome without a chromosome location preference (**Figure 3D**).

### DELs Identification in DHAV-1-Infected DEFs

**Figure 4:**
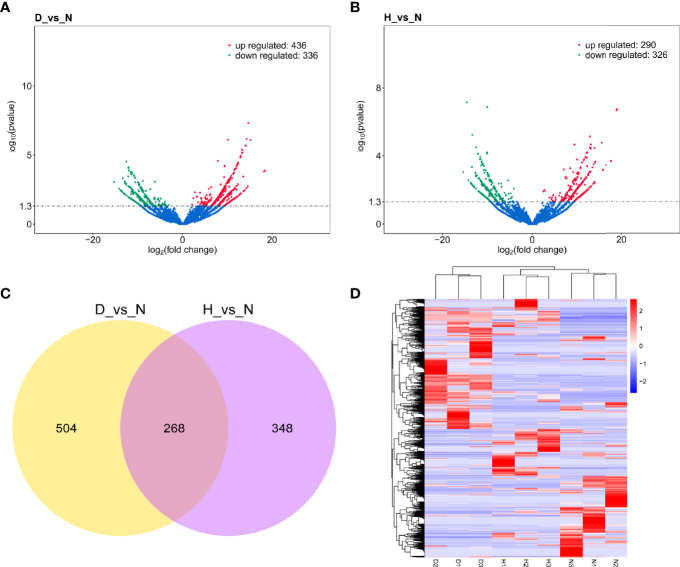
**(A)** Volcano plot of DELs in D-vs-N comparison. **(B)** Volcano plot of DELs in H-vs-N comparison. The vertical line represents a 1.3-fold induction threshold, and the horizontal line represents a cutoff for significance *P* < 0.05. Red points indicated up-regulated transcripts, and green points indicated down-regulated transcripts. **(C)** Venn diagrams depicting the numbers of DELs in D-vs-N comparison and H-vs-N comparison. **(D)** There is hierarchical clustering of DELs in the nine samples (red represents a high expression, and blue represents a low expression).

To identify the high-confidence DELs, the cutoff *P* < 0.05 and | log_2_FC | > 0 were used as a strict screening criterion. The D-vs-N comparison revealed 436 up-regulated and 336 down-regulated DELs (**Figure 4A**, **Table S1**), while the H-vs-N comparison revealed 290 up-regulated and 326 down-regulated DELs (**Figure 4B** and **Table S1**). Notably, the two lncRNA sets shared 268 DELs (**Figure 4C**). The clustering analysis of both DELs showed each group clustered together, demonstrating the low lncRNA profile variability within the same treatment (**Figure 4D**).

### lncRNA Cis- and Trans- Targets Analyses

The target genes of DELs were predicted using cis and trans-regulatory relationships to explore interactive duck lncRNA-mRNA pairs. Regarding cis-action, 772 DELs (D-vs-N comparison) corresponded to 2404 protein-coding genes (**Table S2**), whereas 616 DELs (H-vs-N comparison) neighbored 1970 protein-coding regions (**Table S2**). We also identified 2866 potential target genes for 772 trans-acting lncRNAs (D-vs-N comparison) (**Table S2**) and 1807 potential target genes for 616 trans-acting lncRNAs (H-vs-N comparison) (**Table S2**).

### Functional and Pathway Enrichment Analyses

**Figure 5:**
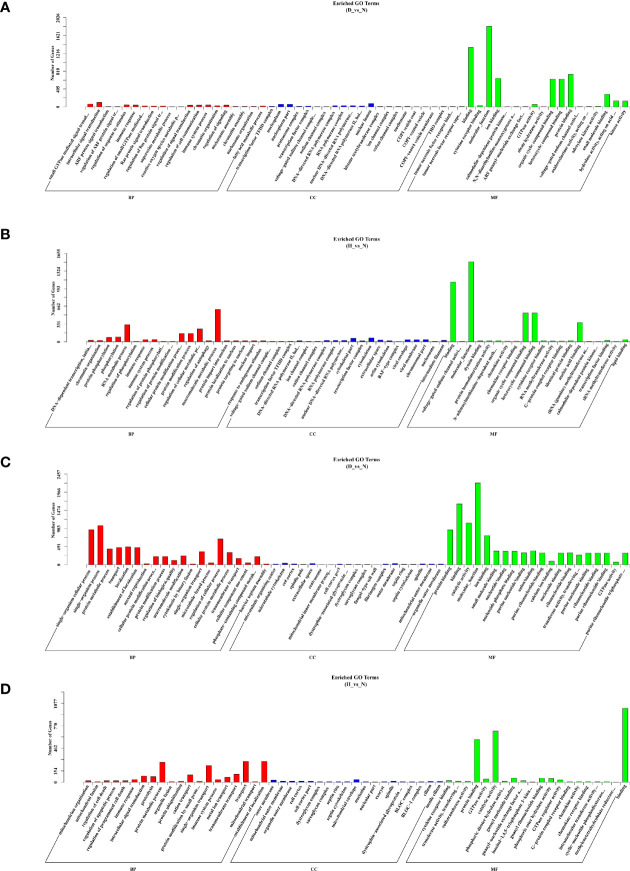
GO enrichment analysis based on DELs’ cis-target genes of D-vs-N group **(A)**, DELs’ cis-target genes of H-vs-N group **(B)**, DELs’ trans-target genes of D-vs-N group **(C)**, and DELs’ trans-target genes of H-vs-N group **(D)**. GO enrichment analysis at each color represents a different biological process. Go terms distribution of target genes under molecular functions, cellular components, and biological processes.

To further characterize the biological functions of the target genes, GO and KEGG analyses were performed. Only the significantly enriched GO terms (*P* < 0.05) were listed derived from cis- and trans-targets (D-vs-N, H-vs-N) (**Table S3**). The GO analysis showed that DELs’ cis-target genes (D-vs-N comparison) were mostly engaged in stimulus-response, signal transduction, phosphorylation, and immune system processes (**Figure 5A**), whereas those of the H-vs-N comparison was mainly engaged in metabolic processes (**Figure 5B**). Moreover, DELs’ trans-target genes (D-vs-N comparison) were mostly involved in biological, cellular, metabolic, single organism processes (**Figure 5C**). Meanwhile, those of the H-vs-N comparison was mainly involved in metabolic processes (**Figure 5D**).

**Figure 6:**
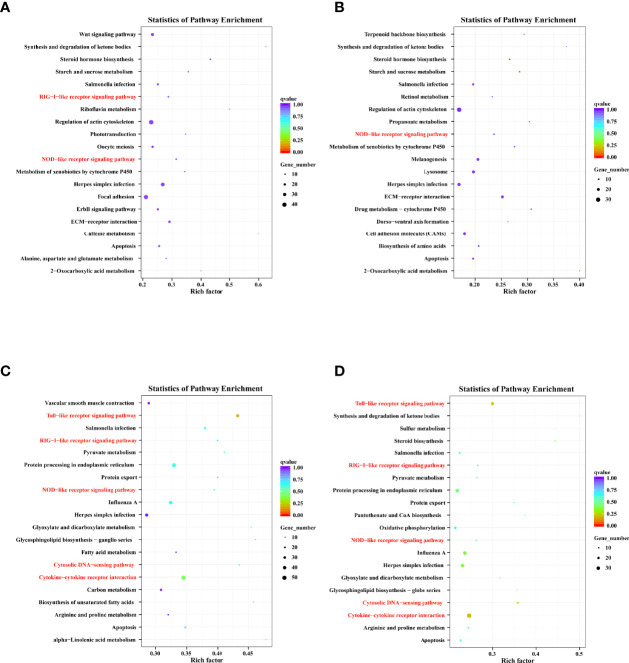
Top 20 KEGG pathway terms based on DELs’ cis-target genes of D-vs-N group **(A)**, DELs’ cis-target genes of H-vs-N group **(B)**, DELs’ trans-target genes of D-vs-N group **(C)**, and DELs’ trans-target genes of H-vs-N group **(D)**.

KEGG enrichment analysis predicted the enriched pathways based on target genes (**Table S4**). The four groups’ top 20 significantly enriched pathways were presented based on a Q-value < 0.05 (**Figure 6**). Furthermore, we noted that the target genes were closely related to the immune system and signal transduction, suggesting that these pathways play an essential role in DHAV-1 progression.

### Co-Expression Networks Construction

**Figure 7:**
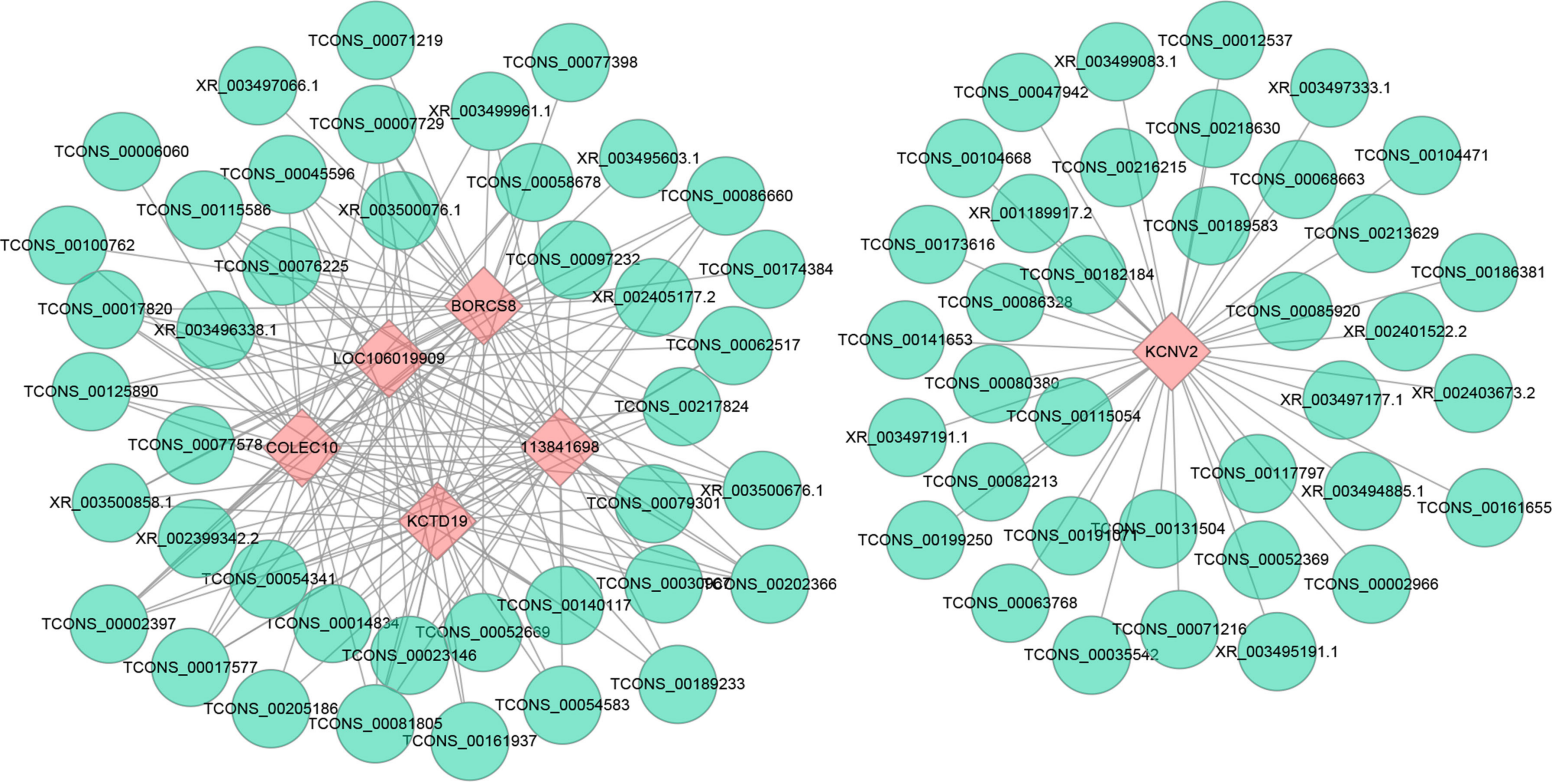
Construction networks of the interaction between DELs and top 6 trans target genes with the largest connections. Blue nodes represent lncRNAs, and pink nodes represent target genes. The line between lncRNAs and target genes indicates an expression pattern correlation.

Multiple trans-acting lncRNAs can simultaneously target some genes. We constructed an interaction network diagram to elucidate their co-expression patterns more clearly (**Figure 7**). The results showed that the top six target genes (which have the most connections, including KCNV2, BORCS8, LOC106019909, LOC113841698, KCTD19, and COLEC10) co-expressed with 38, 35, 33, 33, 31, and 30 DELs, respectively, implying that they play essential roles in DHAV-1 infection.

### Signaling Pathways and Target Genes Involved In Immune Responses and Autophagy Signaling Pathway

We screened crucial immune-related signaling pathways and target genes upon DHAV-1 infection to investigate the virus-induced host innate immune response (**Table S5**). The enriched KEGG pathways were mainly involved in signal transduction and the immune system, including the RIG-I-like receptor signaling pathway (DDX58, IRF7, TRIM25, IFIH1, DHX58), NOD-like receptor signaling pathway (NFκB1, IL6, MAPK14, BIRC2, PIPK2), Cytosolic DNA-sensing pathway (CGAS, DDX58, IRF7, TMEM173, NFκB1), Toll-like receptor signaling pathway (TLR7, TLR3, TLR4, IL8, FADD), JAK-STAT signaling pathway (STAT1, AKT1, SOCS1, STAT6, LOC101801622), MAPK signaling pathway (MAP3K14, ATF4, FGFR4, MAPK10, MAP3K8) and Cytokine-cytokine receptor interaction (CCL20, IL8, TNFRSF8, IL10, IL6).

Furthermore, the number of target genes involved in these immune-related pathways was listed (**Table 3**). It was notable that most cis-target genes were related to the MAPK signaling pathway, whereas most trans-target genes were related to cytokine–cytokine receptor interaction. Notably, the immune-related pathways had more cis- and trans-target genes enriched at 12 hpi (146/213) than at 24 hpi (100/148), indicating that DHAV-1 infection induced a stronger immune response at an early stage.

**Table 3:** The number of the target genes related to innate immunity pathways.

Signaling pathway	cis-target genes of D-vs-N group	trans-target genes of D-vs-N group	cis-target genes of H-vs-N group	trans-target genes of H-vs-N group
NOD-like receptor signaling pathway	12	15	9	10
MAPK signaling pathway	44	47	32	27
Cytokine-cytokine receptor interaction	30	53	18	38
Toll-like receptor signaling pathway	18	36	11	25
RIG-I-like receptor signaling pathway	13	18	8	12
Cytosolic DNA-sensing pathway	6	17	7	14
Jak-STAT signaling pathway	23	27	15	22
Total	146	213	100	148

IFN-stimulated genes (ISGs) participate in antiviral defense. This study identified 100 and 57 potential ISGs among the cis- and trans-target genes in the D-vs-N comparison, while 73 and 39 potential ISGs were found in the cis- and trans-target genes D-vs-H comparison, respectively (**Table 4**). All these genes were previously identified in chickens and ducks (Dai et al., 2020; Yang et al., 2020a). These results revealed that DHAV-1 infection could induce an intensive immune response and activate ISGs expression.

**Table 4:** The number of ISGs (target genes) of different groups.

Groups	The number of ISGs
cis-target genes of D-vs-N group (12hpi)	57
trans-target genes of D-vs-N group (12hpi)	100
cis-target genes of H-vs-N group (24hpi)	39
trans-target genes of H-vs-N group (24hpi)	73

Autophagy is a highly conserved self-eating process in eukaryotic cells. In our study, DELs’ target genes were also involved in important autophagy pathways, including the mTOR signaling pathway (AKT1, HIF1A, RRAGD, IGF1) and the autophagy signaling pathway (ULK1, ULK2, IFNG).

### Experimental Verification of DELs and Corresponding Target Genes

**Figure 8:**
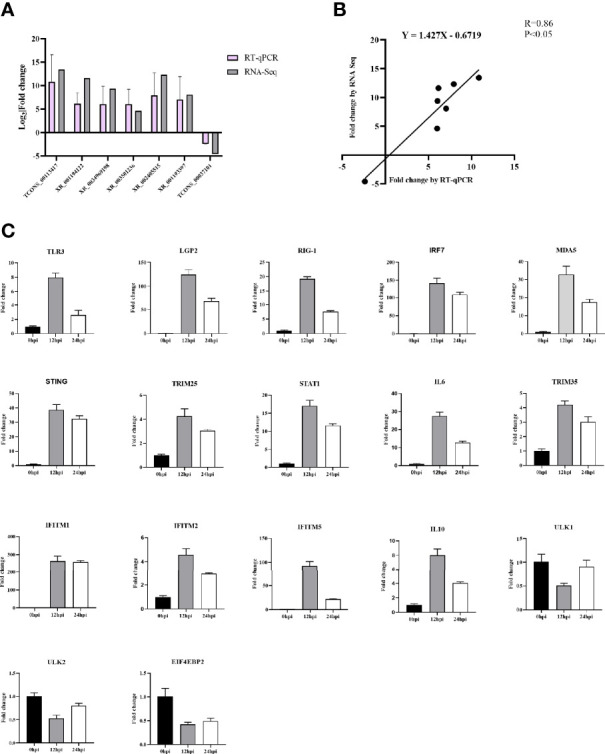
**(A)** Confirmation of DELs by RT-qPCR. **(B)** The Pearson correlation of expression levels of seven DELs between RT-qPCR and RNA-Seq. **(C)** Validation of 18 DELs’ target genes involved in immunity and autophagy signaling pathway after DHAV-1 infection by RT-qPCR.

The crucial immune-related and autophagy-related DELs were quantified using RT-qPCR to verify the relevance of the transcriptomic data. In general, the exhibited expression patterns of RT-qPCR were relatively matched the RNA-Seq data (**Figure 8A**) with a correlation coefficient of 0.86, confirming that the RNA-Seq data were reliable (**Figure 8B**).

We also quantified the 15 immune-related and three autophagy-related target genes’ expression (**Figure 8C**). In the infected group, the 15 immune-related genes were significantly up-regulated than those in the N group, whereas the autophagy-related target genes ULK1, ULK2, and EIF4EBP2 exhibited low expression compared to the control group.

### lncRNA XR_003496198 Inhibits DHAV-1 Replication in DEFs

**Figure 9:**
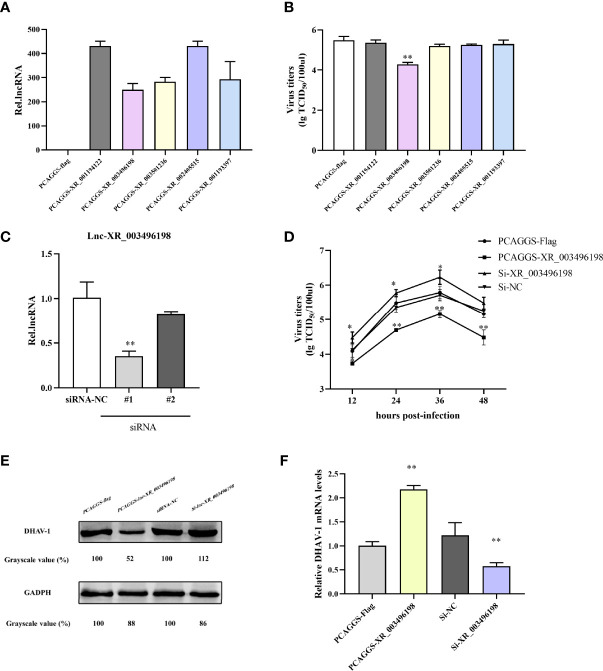
**(A)** RT-qPCR detection of lncRNA overexpression plasmids transfection effects. DEFs were transfected with PCAGGS-lncRNA plasmids or PCAGGS-flag vectors. At 24 hpi, the DEFs were harvested for lncRNA expression detection. **(B)** lncRNA-XR_003496198 can inhibit DHAV-1 replication in DEFs. DEFs were transfected with lncRNA overexpression plasmids and then stimulated with pathogenic DHAV-1. At 24 hpi, the supernatant of DEFs was collected for virus titers detection. **(C)** DEFs were transfected with PCAGGS-XR_003496198, PCAGGS-flag plasmids or si-XR_003496198 or siRNA NC followed infected with DHAV-1. The supernatant of DEFs at different time points was collected for virus titers detection. **(D)** Virus replication was determined by the detection of DHAV-1 proteins. DEFs were transfected with PCAGGS-XR_003496198 or PCAGGS-flag plasmids, si-XR_003496198 or siRNA NC, followed infected with DHAV-1. After 24h infection, DEFs were used to assess the level of DHAV-1 protein replication. * indicated P < 0.05 and ** indicated P < 0.01.

Based on the opinion that lncRNAs accumulated could somehow be involved in regulating virus replication, we screened and cloned five of those mentioned above strongly up-regulated lncRNAs plasmids and transfected them to DEFs to test if lncRNAs regulated DHAV-1. Firstly, we detected the transfection efficiency of the overexpression plasmids, and the results confirmed that the corresponding plasmids were significantly increased the lncRNA expression (**Figure 9A**). Subsequently, we transfected the above plasmids to DEFs, then followed infected with pathogenic DHAV-1. Surprisingly, among theses-lnc-XR_003496198, it was found to inhibit DHAV-1 infection in DEFs (**Figure 9B**). This transcript had no obvious annotation in the NCBI, but after checking its sequence using tools available with the CPC yielded a score of 0.40266 confirmed its noncoding characteristics. CNCI and PFAM also indicated it has almost no coding potential.

We prepared two RNAi pairs and determined their efficiency on the knockdown to further evaluate the antiviral role of endogenous lnc-XR_003496198 during DHAV-1 infection lnc-XR_003496198 using RT-qPCR. The results confirmed that lnc-XR_003496198-RNAi #2 transfection suppressed lnc-XR_003496198 to 10% of control, while the RNAi #1 transfection knockdown lnc-XR_003496198 to 60% of the control (**Figure 9C**). As shown in **Figures 9D**, **F**, knockdown of lnc-XR_003496198 with siRNA significantly promoted the DHAV-1 virus titers. Meanwhile, immunoblotting demonstrated that the expression of DHAV-1 protein was inhibited in DEFs with overexpression of lnc-XR_003496198, whereas increased the DHAV-1 protein in DEFs with reduced expression of lnc-XR_003496198 compared to control cells (**Figure 9E**). Altogether, these results suggest that lnc-XR_003496198 elicited an inhibitory effect on DHAV-1 replication in DEFs.

## Discussion

DVH, mainly caused by DHAV, affects the duck industry by decreasing production efficiency (Tseng et al., 2007). DHAV-1 causes severe acute liver injuries and is the most prevalent serotype in ducklings (Wen et al., 2018). Thus, clarifying the DHAV-1-host interaction molecular mechanisms is urgent. LncRNAs exert vital roles in multiple biological processes such as metabolism and immune modulation (Hombach and Kretz, 2016). Many studies have shown that lncRNAs are critical antiviral effectors during virus-host interactions (Zhu et al., 2020; Wang et al., 2021). However, the role of lncRNAs in DHAV-1-exposed host cells remains unknown. Here, we profiled the expression patterns of lncRNAs in DHAV-1-infected DEFs to elucidate the DHAV-1-induced host immune response. Among all the transcripts, a total of 16589 lncRNAs were found, and 4648 of them were novel (**Table S1**). In DHAV-1-infected DEFs, the most represented lncRNA category was lincRNA, and the majority of the lncRNA lengths ranged from 1000 to 5000 bp. The DEFs lncRNA characteristics were consistent with those of the previous investigation (Lin et al., 2020).

Numerous pathogens can induce and regulate DELs upon infection (Lu et al., 2019; Ma et al., 2019; You et al., 2019). Our transcriptome analysis revealed that DHAV-1 greatly dysregulated lncRNAs, which confirmed that these DELs might be potential DHAV-1 development regulators. Notably, more DELs were detected in the 12 hpi vs. control group (D-vs-N: 772 and H-vs-N: 616), reflecting more intensive changes in the early stages of DHAV-1 infection.

The most important pattern for lncRNAs to function on neighboring genes is mainly in cis- or trans- manners (Bai et al., 2015; Yamada, 2017; Gao et al., 2020). Consequently, we predicted the DELs target genes and performed GO and KEGG analysis to further explore the regulatory pathways potentially involved in DHAV-1 infection. According to functional annotation, infection enriched numerous innate immune response target genes. Therefore, most follow-up studies have focused on the immune-related target genes in response to DHAV-1, including the key pattern recognition receptors (PRRs), downstream pathways of PRRs, and effector molecules, such as cytokines and ISGs.

Innate PRRs play important defensive roles against viral infections (Zhao et al., 2016). We found KEGG pathway annotations for various target genes involved in the Toll-like receptor (TLR) signaling, NOD-like receptor signaling, RIG-I-like receptor signaling, and cytosolic DNA sensing pathways. For example, a panel of TLRs including TLR3, TLR4, and TLR7 (targeted by lnc-XR_001188764.3, lnc-TCONS_00161164; lnc-TCONS_00124241; lnc-TCONS_00142332, lnc-TCONS_00002813, lnc-XR_002398570.2) was involved in the TLR signaling pathway. TLR3 is an essential PRR for monitoring viral double-stranded RNA (dsRNA), and lncRNAs are potential regulators in TLR3-driven antiviral responses (Murphy and Medvedev, 2016). For example, lnc-NEAT1 facilitates TLR3-stimulated CXCL8 expression in HeLa and A549 cells by binding to SFPQ (a transcriptional CXCL8 inhibitor), leading to SFPQ transposition away from the CXCL8 promotor (Boo and Yang, 2010). Additionally, the up-regulation of TLR3 occurs during multiple virus infections such as Muscovy Duck Reovirus (MDRV), Duck Tembusu virus (DTMUV), and Zika virus (ZIKV) (Bendelja et al., 2010; Zhang et al., 2015; Dang et al., 2016). Similarly, our results showed that DHAV-1 greatly increases TLR3 expression.

The retinoic acid-inducible gene-I-like receptor (RLR) family consists of RIG-I, MDA5, and LGP2. RIG-I and MDA5 primarily recognize cytosolic ssRNA and dsRNA (Kato et al., 2006), and they share similar protein structures, notably an N-terminal tandem caspase activation and recruitment domain (CARD), a central DExD/H box RNA helicase domain, and a C-terminal repressor domain (Loo and Gale, 2011). LGP2 lacks an N-terminal CARD, and its exact role is still controversial (Saito et al., 2007). Upon ligand recognition, RIG-I and MDA5 recruit the MAVS adaptor protein *via* CARD–CARD interaction and activate the downstream TBK1 and inducible IKKs. Signal transduction activates NF-κB and IRF7, leading to their translocation to the nucleus and promoting IFN-I expression (Loo and Gale, 2011). In this study, several components of the RLR signaling pathway, including DDX58 (RIG-I, targeted by six DELs), IFIH1 (MDA5, targeted by eight DELs), IRF7 (targeted by two DELs), TRIM25 (targeted by two DELs), and DHX58 (LGP2, targeted by five DELs) were all predicted based on the KEGG analysis and these genes expression were significantly up-regulated from RT-qPCR analysis. Moreover, accumulating evidence suggests that lncRNAs play an important role in the RLR signaling pathway. For instance, lnc-ITPRIP-1 up-regulation can positively regulate the RLR signaling pathway by targeting MDA5 and suppressing HCV replication (Xie et al., 2018). Similarly, lnc-NEAT1 inhibits HIV infection by positively regulating RIG-I-DDX60-mediated IFN responses (Ma et al., 2017). Moreover, lnc-zc3h7a and TRIM25 synergize to block virus attacks by enhancing TRIM25-mediated K63-linked ubiquitination of RIG-I, which is required for downstream antiviral signaling activation (Lin et al., 2019). Therefore, we speculate that these DELs could target crucial RLR signaling pathway genes, thereby defending against DHAV-1 infection.

Interestingly, DHAV-1 treatment up-regulated cGAS (targeted by lnc-XR_001193465.3) expression. cGAS plays a role in the cytosolic DNA receptor signaling pathway, and it is generally accepted that DNA viruses activate this pathway. Indeed, RNA viruses can also manipulate DNA sensors (Liu et al., 2017a; Abe et al., 2019). It has been proven that DTMUV-encoded NS2A protein disrupts du-STING-dependent antiviral cellular defenses by binding to du-STING (Zhang et al., 2020). The potential interaction between the viral protein and cytosolic DNA sensing pathway facilitates the virus’ escaping strategy. However, interactions between this pathway and DHAV-1 remain unclear and require in-depth research.

PRRs independently activated the JAK-STAT and the MAPK signaling pathways. The D-vs-N and H-vs-N groups contain 27 and 22 trans-target genes participating in the JAK-STAT signaling pathway, respectively, and 47 and 27 trans-target genes involved in the MAPK signaling pathway. STAT1 is a critical transcription factor involved in signal transduction (Marié et al., 2018) targeted by the lnc-TCONS_00113417 in our study, and its expression was significantly up-regulated. However, many viruses associate with STAT1 to antagonize or inhibit the IFN-triggered JAK-STAT signal transduction cascade from evading the host’s innate immune responses. For instance, TCBV NS4A inhibits STAT1/STAT2 phosphorylation and dimerization to block nuclear translocation and transcriptional regulation (Yang et al., 2020b). Besides, DEV evades innate immunity clearance thanks to its UL47 protein, which interacts with STAT1 to suppress signal transduction (Dar et al., 2005). Therefore, we speculated that DHAV-1 infection could activate the JAK-STAT signaling pathway efficiently.

Cytokines are soluble extracellular mediators such as chemokines, IFN, interleukins (IL), lymphokines, and tumor necrosis factors, which have important roles in immune response activation. IL-6 is a pleiotropic cytokine that regulates hematopoiesis and immune and inflammatory responses and contributes to T and B cell activation, proliferation, and differentiation (Chen et al., 2020). IL-8, also known as CXCL8, is an important immune response mediator produced by macrophages and peripheral blood mononuclear cells (Zou et al., 2016). In contrast, IL-10 can inhibit the synthesis and release of immune mediators and block antigen presentation to T cells and macrophages, thereby reducing the inflammatory response (Liu et al., 2013). The KEGG analysis predicted that DHAV-1 would affect many important cytokines and cytokine receptor genes, including IL-6, IL-8, and IL-10. Besides, the lnc-TCONS_00113417, whose target gene was IL-8, exhibited the second highest expression level change (12.4-fold) among all DELs, and the expression of both IL-6 and IL-10 were up-regulated, indicating that the protective response occurred during DHAV-1 infection. Compared with cytokine changes in DHAV-3-infected duck livers, IL-6 and IL-10 expression increased significantly (Zhang et al., 2018). Therefore, we assumed that DHAV-1 and DHAV-3 induce similar changes in immunological characteristics.

Type "I" signaling pathway activation increases ISG expression, which plays an important role in antiviral defense, antiproliferative activities, and adaptive immunity stimulation (Sen and Sarkar, 2007). In our study, we respectively identified 100 and 57 potential ISGs in the cis- and trans-target genes in the D-vs-N group and 73 and 39 in the D-vs-H group (**Table S6**). These include interferon-induced transmembrane (IFITM) proteins, such as IFITM1, IFITM3, and IFITM5, which generally play a part in antiviral immunity. IFITM proteins restrict virus replication by blocking viral entry and preventing virus fusion with the late endosome or lysosome and penetration into the cytoplasm (Huang et al., 2011; Li et al., 2017). Recently, many tripartite motif family proteins have been confirmed to be ISGs, which regulate the innate immune response. For example, TRIM25 has a role in TRAF6-mediated NF-κB activation and positively regulates the RLR signaling pathway (Lee et al., 2015). Meanwhile, TRIM35 defends the host against the influenza A virus by forming circuits among TRIM35, TRAF3, and influenza A virus’ PB2 (Sun et al., 2020). We confirmed the up-regulation of TRIM25, TRIM35, and TRIM66 (targeted by lnc-TCONS_00182498, lnc-XR_003500542.1, lnc-TCONS_00012417, lnc-TCONS_00191851; lnc-TCONS_00049845, lnc-XR_002401149.2; lnc-XR_003498594.1) using RT-qPCR. Hence, these genes may be essential to the response against DHAV-1 infection.

Besides the target genes participating in the innate immune response during virus attack, our transcriptome sequencing data also proved that some DELs targeted ULK1, ULK2, ATG3, and ATG12, which have been reported to be involved in autophagy (Levine and Kroemer, 2019). Moreover, multiple DELs could simultaneously target genes, implying important roles in DHAV-1 infection. In our study, six target genes (KCNV2, LOC106019909, 113841698, BORCS8, COLE10, and KCTD19) were co-expressed with 38, 33, 33, 35, 30, and 31 DELs, respectively. Thus, the lncRNA-mRNA crosstalk provided more considerable information and confirmed the complexity of the gene regulatory network.

LncRNAs are the critical regulators between virus and host and are involved in the biological process ranging from control of viral and host genes, regulation of stability and translation of mRNAs, and manuscript of host antiviral response (Carpenter et al., 2013; Liu and Ding, 2017). Our study found that lnc-XR_003496198, which has no obvious coding potential, is located at chromosome 3. Increased the expression of lnc-XR_003496198 can repress the replication of DHAV-1 at transcript and protein levels, while knockdown of it can remarkably promote DHAV-1 proliferation. However, the exact regulatory mechanisms of lnc-XR_003496198 have not been investigated and require further research.

In summary, we reported the first comprehensive description of lncRNA profiles upon DHAV-1 infection and screened multiple lncRNAs and putative target genes involved in innate immunity and autophagy. Moreover, seven lncRNAs were significantly induced upon DHAV-1 infection were determined, and the results showed that XR_003496198 overexpression in DEFs led to a reduced DHAV-1 transcription and translation. These findings provide valuable transcriptomic information for better understanding the immune response of host cells. Further investigations will focus on explaining specific regulation mechanisms of these lncRNAs and searching for a potential therapeutic target against Picornavirus-related diseases.

## Data Availability Statement

The datasets presented in this study can be found in online repositories. The names of the repository/repositories and accession number(s) can be found in the article/**Supplementary Material**.

## Ethics Statement

The animal study was reviewed and approved by the Animal Care and Use Committee of Shandong Agricultural University (# SDAUA-2018-045).

## Author Contributions

Conceptualization, JH and NS. Methodology, JH. Software, NS and YJ. Validation, NS, YJ, and HY. Formal analysis, JW. Investigation, GX and YZ. Resources, ZX. Data curation, NS. Writing—original draft preparation, NS and JH. Writing—review and editing, NS, JH and SJ. Visualization, NS, YZ. Supervision, NS, JH and SJ. All authors contributed to the article and approved the submitted version.

## Funding

This study was supported by grants from the National Natural Science Foundation of China (31772754), Shandong Modern Agricultural Technology & Industry System, China (SDAIT-11-15), and Funds of Shandong. “Double Tops” Program, China (SYL2017YSTD11).

## Conflict of Interest

Author JH was employed by the company Shandong GreenBlue Biotechnology Co. Ltd.

The remaining authors declare that the research was conducted in the absence of any commercial or financial relationships that could be construed as a potential conflict of interest.

## Publisher’s Note

All claims expressed in this article are solely those of the authors and do not necessarily represent those of their affiliated organizations, or those of the publisher, the editors and the reviewers. Any product that may be evaluated in this article, or claim that may be made by its manufacturer, is not guaranteed or endorsed by the publisher.

## References

[B1] Abe T. Marutani Y. Shoji I. (2019). Cytosolic DNA-Sensing Immune Response and Viral Infection. Microbiol. Immunol. 63 (2), 51–64. doi: 10.1111/1348-0421.12669 30677166 PMC7168513

[B2] Agarwal S. Vierbuchen T. Ghosh S. Chan J. Jiang Z. Kandasamy R. K.. (2020). The Long Noncoding RNA LUCAT1 is a Negative Feedback Regulator of Interferon Responses in Humans. Nat. Commun. 11 (1), 6348. doi: 10.1038/s41467-020-20165-5 33311506 PMC7733444

[B3] Agliano F. Rathinam V. A. Medvedev A. E. Vanaja S. K. Vella A. T. (2019). Long Noncoding RNAs in Host–Pathogen Interactions. Trends Immunol. 40 (6), 492–510. doi: 10.1016/j.it.2019.04.001 31053495 PMC6556373

[B4] Bai Y. Dai X. Harrison A. P. Chen M. (2015). RNA Regulatory Networks in Animals and Plants: A Long Noncoding RNA Perspective. Brief Funct. Genomics 14 (2), 91–101. doi: 10.1093/bfgp/elu017 24914100

[B5] Bendelja K. Vojvoda V. Aberle N. Cepin-Bogovic J. Gagro A. Mlinaric-Galinovic G.. (2010). Decreased Toll-Like Receptor 8 Expression and Lower TNF-Alpha Synthesis in Infants With Acute RSV Infection. Respir. Res. 11 (1), 143. doi: 10.1186/1465-9921-11-143 20946625 PMC2974671

[B6] Boo K.-H. Yang J.-S. (2010). Intrinsic Cellular Defenses Against Virus Infection by Antiviral Type I Interferon. Yonsei Med. J. 51 (1), 9–17. doi: 10.3349/ymj.2010.51.1.9 20046508 PMC2799977

[B7] Carninci P. Kasukawa T. Katayama S. Gough J. Frith M. C. Maeda N.. (2005). The Transcriptional Landscape of the Mammalian Genome. Science 309 (5740), 1559–1563. doi: 10.1126/science.1112014 16141072

[B8] Carpenter S. Aiello D. Atianand M. K. Ricci E. P. Gandhi P. Hall L. L.. (2013). A Long Noncoding RNA Mediates Both Activation and Repression of Immune Response Genes. Science 341 (6147), 789–792. doi: 10.1126/science.1240925 23907535 PMC4376668

[B9] Chalmers W. S. K. Woolcock P. R. (1984). The Effect of Animal Sera on Duck Hepatitis Virus. Avian Pathol. 13 (4), 727–732. doi: 10.1080/03079458408418569 18766882

[B10] Cheng Y. Liu Y. Shi S. Niu Q. Zhu W. Wang Z.. (2019). Functional Characterization of Duck STING in IFN-β Induction and Anti-H9N2 Avian Influenza Viruses Infections. Front. Immunol. 10. doi: 10.3389/fimmu.2019.02224 PMC6759682 31620135

[B11] Chen Y. G. Satpathy A. T. Chang H. Y. (2017). Gene Regulation in the Immune System by Long Noncoding RNAs. Nat. Immunol. 18 (9), 962–972. doi: 10.1038/ni.3771 28829444 PMC9830650

[B12] Chen S. Tang Y. Yang C. Li K. Huang X. Cao J. (2020). Silencing CDC25A Inhibits the Proliferation of Liver Cancer Cells by Downregulating IL−6 *In Vitro* and *In Vivo* . Int. J. Mol. Med. 45 (3), 743–752. doi: 10.3892/ijmm.2020.4461 31922225 PMC7015122

[B13] Chen S. Wang T. Liu P. Yang C. Wang M. Jia R.. (2019). Duck Interferon Regulatory Factor 7 (IRF7) can Control Duck Tembusu Virus (DTMUV) Infection by Triggering Type I Interferon Production and its Signal Transduction Pathway. Cytokine 113, 31–38. doi: 10.1016/j.cyto.2018.06.001 29885990

[B14] Dai M. Xie T. Liao M. Zhang X. Feng M. (2020). Systematic Identification of Chicken Type I, II and III Interferon-Stimulated Genes. Vet. Res. 51 (1), 70. doi: 10.1186/s13567-020-00793-x 32448397 PMC7245633

[B15] Dang J. Tiwari S. K. Lichinchi G. Qin Y. Patil V. S. Eroshkin A. M.. (2016). Zika Virus Depletes Neural Progenitors in Human Cerebral Organoids Through Activation of the Innate Immune Receptor Tlr3. Cell Stem Cell. 19 (2), 258–265. doi: 10.1016/j.stem.2016.04.014 27162029 PMC5116380

[B16] Dar A. Munir S. Vishwanathan S. Manuja A. Griebel P. Tikoo S.. (2005). Transcriptional Analysis of Avian Embryonic Tissues Following Infection With Avian Infectious Bronchitis Virus. Virus Res. 110 (1-2), 41–55. doi: 10.1016/j.virusres.2005.01.006 15845254 PMC7114260

[B17] Ding C. Zhang D. (2007). Molecular Analysis of Duck Hepatitis Virus Type 1. Virology 361 (1), 9–17. doi: 10.1016/j.virol.2007.01.007 17300822

[B18] El-Gebali S. Mistry J. Bateman A. Eddy S. R. Luciani A. Potter S. C.. (2019). doi: 10.1093/nar/gky995

[B19] Finn R. D. Bateman A. Clements J. Coggill P. Eberhardt R. Y. Eddy S. R.. (2014). Pfam: The Protein Families Database. Nucleic Acids Res. 30 (1), 276–280. doi: 10.1093/nar/gkt1223

[B20] Fu Y. Pan M. Wang X. Xu Y. Yang H. Zhang D. (2008). Molecular Detection and Typing of Duck Hepatitis A Virus Directly From Clinical Specimens. Vet. Microbiol. 131 (3-4), 247–257. doi: 10.1016/j.vetmic.2008.03.011 18462894

[B21] Gao J. Chen J. Si X. Xie Z. Zhu Y. Zhang X.. (2012). Genetic Variation of the VP1 Gene of the Virulent Duck Hepatitis A Virus Type 1 (DHAV-1) Isolates in Shandong Province of China. Virol. Sin. 27 (4), 248–253. doi: 10.1007/s12250-012-3255-8 22899433 PMC8218140

[B22] Gao C. Sun J. Dong Y. Wang C. Xiao S. Mo L.. (2020). Comparative Transcriptome Analysis Uncovers Regulatory Roles of Long Noncoding RNAs Involved in Resistance to Powdery Mildew in Melon. BMC Genomics 21 (1), 125. doi: 10.1186/s12864-020-6546-8 32024461 PMC7003419

[B23] Ghosh S. Chan C.-K. K. (2016). Analysis of RNA-Seq Data Using TopHat and Cufflinks. Methods Mol. Biol. 1374, 339–361. doi: 10.1007/978-1-4939-3167-5_18 26519415

[B24] Hao Q. Wang Z. Wang Q. Chen B. Qian H. Liu X.. (2021). Identification and Characterization of lncRNA AP000253 in Occult Hepatitis B Virus Infection. Virol. J. 18 (1), 125. doi: 10.1186/s12985-021-01596-y 34112188 PMC8194241

[B25] Hombach S. Kretz M. (2016). Noncoding RNAs: Classification, Biology and Functioning. Adv. Exp. Med. Biol. 937, 3–17. doi: 10.1007/978-3-319-42059-2_1 27573892

[B26] Huang I. C. Bailey C. C. Weyer J. L. Radoshitzky S. R. Becker M. M. Chiang J. J.. (2011). Distinct Patterns of IFITM-Mediated Restriction of Filoviruses, SARS Coronavirus, and Influenza A Virus. PloS Pathog. 7 (1), e1001258. doi: 10.1371/journal.ppat.1001258 21253575 PMC3017121

[B27] Hu J. Yang D. Wang H. Li C. Zeng Y. Chen W. (2016). CpG Oligodeoxynucleotides Induce Differential Cytokine and Chemokine Gene Expression Profiles in Dapulian and Landrace Pigs. Front. Microbiol. 7, 1992. doi: 10.3389/fmicb.2016.01992 28018321 PMC5156958

[B28] Iyer M. K. Niknafs Y. S. Malik R. Singhal U. Sahu A. Hosono Y.. (2015). The Landscape of Long Noncoding RNAs in the Human Transcriptome. Nat. Genet. 47 (3), 199–208. doi: 10.1038/ng.3192 25599403 PMC4417758

[B29] Jin X. Zhang W. Zhang W. Gu C. Cheng G. Hu X. (2008). Identification and Molecular Analysis of the Highly Pathogenic Duck Hepatitis Virus Type 1 in Hubei Province of China. Res. Vet. Sci. 85 (3), 595–598. doi: 10.1016/j.rvsc.2008.01.001 18486957

[B30] Kato H. Takeuchi O. Sato S. Yoneyama M. Yamamoto M. Matsui K.. (2006). Differential Roles of MDA5 and RIG-I Helicases in the Recognition of RNA Viruses. Nature 441 (7089), 101–105. doi: 10.1038/nature04734 16625202

[B31] Kim D. Langmead B. Salzberg S. L. (2015). HISAT: A Fast Spliced Aligner With Low Memory Requirements. Nat. Methods 12 (4), 357–360. doi: 10.1038/nmeth.3317 25751142 PMC4655817

[B32] Kong L. Zhang Y. Ye Z.-Q. Liu X.-Q. Zhao S.-Q. Wei L.. (2007). CPC: Assess the Protein-Coding Potential of Transcripts Using Sequence Features and Support Vector Machine. Nucleic Acids Res. 35 (Web Server issue), W345–W349. doi: 10.1093/nar/gkm391 17631615 PMC1933232

[B33] Lan J. Zhang R. Yu H. Wang J. Xue W. Chen J.. (2019). Quantitative Proteomic Analysis Uncovers the Mediation of Endoplasmic Reticulum Stress-Induced Autophagy in DHAV-1-Infected DEF Cells. Int. J. Mol. Sci. 20 (24), 6160. doi: 10.3390/ijms20246160 PMC6940786 31817666

[B34] Lee N.-R. Kim H.-I. Choi M.-S. Yi C.-M. Inn K.-S. (2015). Regulation of MDA5-MAVS Antiviral Signaling Axis by TRIM25 Through TRAF6-Mediated NF-κb Activation. Mol. Cells 38 (9), 759–764. doi: 10.14348/molcells.2015.0047 26299329 PMC4588718

[B35] Levine B. Kroemer G. (2019). Biological Functions of Autophagy Genes: A Disease Perspective. Cell 176 (1-2), 11–42. doi: 10.1016/j.cell.2018.09.048 30633901 PMC6347410

[B36] Li Z. Chao T. C. Chang K. Y. Lin N. Patil V. S. Shimizu C.. (2014). The Long Noncoding RNA THRIL Regulates TNFalpha Expression Through its Interaction With hnRNPL. Proc. Natl. Acad. Sci. U.S.A. 111 (3), 1002–1007. doi: 10.1073/pnas.1313768111 24371310 PMC3903238

[B37] Lin S. L. Cong R. C. Zhang R. H. Chen J. H. Xia L. L. Xie Z. J.. (2016). Circulation and *In Vivo* Distribution of Duck Hepatitis A Virus Types 1 and 3 in Infected Ducklings. Arch. Virol. 161 (2), 405–416. doi: 10.1007/s00705-015-2648-z 26597185

[B38] Lin H. Jiang M. Liu L. Yang Z. Ma Z. Liu S.. (2019). The Long Noncoding RNA Lnczc3h7a Promotes a TRIM25-Mediated RIG-I Antiviral Innate Immune Response. Nat. Immunol. 20 (7), 812–823. doi: 10.1038/s41590-019-0379-0 31036902

[B39] Lin Y. Yang J. He D. Li X. Li J. Tang Y.. (2020). Differently Expression Analysis and Function Prediction of Long Non-Coding RNAs in Duck Embryo Fibroblast Cells Infected by Duck Tembusu Virus. Front. Immunol. 11, 1729. doi: 10.3389/fimmu.2020.01729 32849615 PMC7417515

[B40] Li P. Shi M.-L. Shen W.-L. Zhang Z. Xie D.-J. Zhang X.-Y.. (2017). Coordinated Regulation of IFITM1, 2 and 3 Genes by an IFN-Responsive Enhancer Through Long-Range Chromatin Interactions. Biochim. Biophys. Acta Gene Regul. Mech. 1860 (8), 885–893. doi: 10.1016/j.bbagrm.2017.05.003 28511927 PMC7102783

[B41] Liu W. Ding C. (2017). Roles of LncRNAs in Viral Infections. Front. Cell Infect. Microbiol. 7. doi: 10.3389/fcimb.2017.00205 PMC5445353 28603696

[B42] Liu K. Kaur R. Almudevar A. Pichichero M. E. (2013). Higher Serum Levels of Interleukin 10 Occur at Onset of Acute Otitis Media Caused by Streptococcus Pneumoniae Compared to Haemophilus Influenzae and Moraxella Catarrhalis. Laryngoscope 123 (6), 1500–1505. doi: 10.1002/lary.23973 23404508 PMC3966296

[B43] Liu Y. Lin R. Olagnier D. (2017a). RIGulation of STING Expression: At the Crossroads of Viral RNA and DNA Sensing Pathways. Inflammation Cell Signal. 4 (1), e1491. doi: 10.14800/ics.1491 PMC5298905 28191486

[B44] Livak K. J. Schmittgen T. D. (2001). Analysis of Relative Gene Expression Data Using Real-Time Quantitative PCR and the 2–ΔΔct Method. Methods 25 (4), 402–408. doi: 10.1006/meth.2001.1262 11846609

[B45] Li N. Wang Y. Li R. Liu J. Zhang J. Cai Y.. (2015). Immune Responses of Ducks Infected With Duck Tembusu Virus. Front. Microbiol. 6. doi: 10.3389/fmicb.2015.00425 PMC4424876 26005441

[B46] Loo Y.-M. Gale M. (2011). Immune Signaling by RIG-I-Like Receptors. Immunity 34 (5), 680–692. doi: 10.1016/j.immuni.2011.05.003 21616437 PMC3177755

[B47] Lu C. Xing Y. Cai H. Shi Y. Liu J. Huang Y. (2019). Identification and Analysis of Long Noncoding RNAs in Response to H5N1 Influenza Viruses in Duck (Anas Platyrhynchos). BMC Genomics 20 (1), 36. doi: 10.1186/s12864-018-5422-2 30634898 PMC6330444

[B48] Ma H. Han P. Ye W. Chen H. Zheng X. Cheng L.. (2017). The Long Noncoding RNA NEAT1 Exerts Antihantaviral Effects by Acting as Positive Feedback for RIG-I Signaling. J. Virol. 91 (9), e02250–e02216. doi: 10.1128/JVI.02250-16 28202761 PMC5391460

[B49] Marié I. J. Chang H.-M. Levy D. E. (2018). HDAC Stimulates Gene Expression Through BRD4 Availability in Response to IFN and in Interferonopathies. J. Exp. Med. 215 (12), 3194–3212. doi: 10.1084/jem.20180520 30463877 PMC6279398

[B50] Ma X. Zhao X. Wang K. Tang X. Guo J. Mi M.. (2019). Identification and Analysis of Long Noncoding RNAs That are Involved in Inflammatory Process in Response to Transmissible Gastroenteritis Virus Infection. BMC Genomics 20 (1), 806. doi: 10.1186/s12864-019-6156-5 31684870 PMC6829948

[B51] Murphy M. B. Medvedev A. E. (2016). Long Noncoding RNAs as Regulators of Toll-Like Receptor Signaling and Innate Immunity. J. Leukoc. Biol. 99 (6), 839–850. doi: 10.1189/jlb.2ru1215-575r 26965636 PMC6608019

[B52] Ou X. Mao S. Cao J. Cheng A. Wang M. Zhu D.. (2017). Comparative Analysis of Virus-Host Interactions Caused by a Virulent and an Attenuated Duck Hepatitis A Virus Genotype 1. PloS One 12 (6), e0178993. doi: 10.1371/journal.pone.0178993 28614378 PMC5470708

[B53] Pertea M. Kim D. Pertea G. M. Leek J. T. Salzberg S. L. (2016). Transcript-Level Expression Analysis of RNA-Seq Experiments With HISAT, StringTie and Ballgown. Nat. Protoc. 11 (9), 1650–1667. doi: 10.1038/nprot.2016.095 27560171 PMC5032908

[B54] Qian W. Wei X. Li Y. Guo K. Zou Z. Zhou H.. (2018). Duck Interferon Regulatory Factor 1 Acts as a Positive Regulator in Duck Innate Antiviral Response. Dev. Comp. Immunol. 78, 1–13. doi: 10.1016/j.dci.2017.09.004 28890139

[B55] Saito T. Hirai R. Loo Y.-M. Owen D. Johnson C. L. Sinha S. C.. (2007). Regulation of Innate Antiviral Defenses Through a Shared Repressor Domain in RIG-I and LGP2. Proc. Natl. Acad. Sci. U.S.A. 104 (2), 582–587. doi: 10.1073/pnas.0606699104 17190814 PMC1766428

[B56] Sen G. C. Sarkar S. N. (2007). The Interferon-Stimulated Genes: Targets of Direct Signaling by Interferons, Double-Stranded RNA, and Viruses. Curr. Top. Microbiol. Immunol. 316, 233–250. doi: 10.1007/978-3-540-71329-6_12 17969451

[B57] Song C. Yu S. Duan Y. Hu Y. Qiu X. Tan L.. (2014). Effect of Age on the Pathogenesis of DHV-1 in Pekin Ducks and on the Innate Immune Responses of Ducks to Infection. Arch. Virol. 159 (5), 905–914. doi: 10.1007/s00705-013-1900-7 24162826

[B58] Stark R. Grzelak M. Hadfield J. (2019). RNA Sequencing: The Teenage Years. Nat. Rev. Genet. 20 (11), 631–656. doi: 10.1038/s41576-019-0150-2 31341269

[B59] Sun N. Jiang L. Ye M. Wang Y. Wang G. Wan X.. (2020). TRIM35 Mediates Protection Against Influenza Infection by Activating TRAF3 and Degrading Viral PB2. Protein Cell. 11 (12), 894–914. doi: 10.1007/s13238-020-00734-6 32562145 PMC7719147

[B60] Sun L. Luo H. Bu D. Zhao G. Yu K. Zhang C.. (2013). Utilizing Sequence Intrinsic Composition to Classify Protein-Coding and Long Noncoding Transcripts. Nucleic Acids Res. 41 (17), e166. doi: 10.1093/nar/gkt646 23892401 PMC3783192

[B61] Tseng C. H. Knowles N. J. Tsai H. J. (2007). Molecular Analysis of Duck Hepatitis Virus Type 1 Indicates That it Should be Assigned to a New Genus. Virus Res. 123 (2), 190–203. doi: 10.1016/j.virusres.2006.09.007 17067712

[B62] Wang M. Chai L. Liang S. Lv J. Yang L. Qu S.. (2020). Fetal Calf Serum Exerts an Inhibitory Effect on Replication of Duck Hepatitis A Virus Genotype 1 in Duck Embryo Fibroblast Cells. Viruses 12 (1), 80. doi: 10.3390/v12010080 PMC7019637 31936491

[B63] Wang Y. Huo Z. Lin Q. Lin Y. Chen C. Huang Y.. (2021). Positive Feedback Loop of Long Noncoding RNA OASL-IT1 and Innate Immune Response Restricts the Replication of Zika Virus in Epithelial A549 Cells. J. Innate Immun. 13 (3), 1–15. doi: 10.1159/000513606 33626545 PMC8138224

[B64] Wen X. Zhu D. Cheng A. Wang M. Chen S. Jia R.. (2018). Molecular Epidemiology of Duck Hepatitis a Virus Types 1 and 3 in Chin-2015. Transbound Emerg. Dis. 65 (1), 10–15. doi: 10.1111/tbed.12741 29076646

[B65] Xie Q. Chen S. Tian R. Huang X. Deng R. Xue B.. (2018). Long Noncoding RNA ITPRIP-1 Positively Regulates the Innate Immune Response Through Promotion of Oligomerization and Activation of MDA5. J. Virol. 92 (17), e00507–e00518. doi: 10.1128/JVI.00507-18 29899107 PMC6096792

[B66] Xie J. Wang M. Cheng A. Zhao X.-X. Liu M. Zhu D.. (2019). DHAV-1 Inhibits Type I Interferon Signaling to Assist Viral Adaption by Increasing the Expression of SOCS3. Front. Immunol. 10. doi: 10.3389/fimmu.2019.00731 PMC6465609 31024559

[B67] Yamada M. (2017). Functions of Long Intergenic Noncoding (Linc) RNAs in Plants. J. Plant Res. 130 (1), 67–73. doi: 10.1007/s10265-016-0894-0 27999969

[B68] Yang L. Li J. Bi Y. Xu L. Liu W. (2012). Development and Application of a Reverse Transcription Loop-Mediated Isothermal Amplification Method for Rapid Detection of Duck Hepatitis A Virus Type 1. Virus Genes 45 (3), 585–589. doi: 10.1007/s11262-012-0798-6 22869367 PMC7088793

[B69] Yang Y. Li L. Liu X. Jiang M. Zhao J. Li X.. (2020a). Quantitative Proteomic Analysis of Duck Embryo Fibroblasts Infected With Novel Duck Reovirus. Front. Vet. Sci. 7. doi: 10.3389/fvets.2020.577370 PMC7738351 33344524

[B70] Yang Q. You J. Zhou Y. Wang Y. Pei R. Chen X.. (2020b). Tick-Borne Encephalitis Virus NS4A Ubiquitination Antagonizes Type I Interferon-Stimulated STAT1/2 Signalling Pathway. Emerg. Microbes Infect. 9 (1), 714–726. doi: 10.1080/22221751.2020.1745094 32196427 PMC7170394

[B71] Yao R.-W. Wang Y. Chen L.-L. (2019). Cellular Functions of Long Noncoding RNAs. Nat. Cell Biol. 21 (5), 542–551. doi: 10.1038/s41556-019-0311-8 31048766

[B72] You Z. Zhang Q. Liu C. Song J. Yang N. Lian L. (2019). Integrated Analysis of lncRNA and mRNA Repertoires in Marek’s Disease Infected Spleens Identifies Genes Relevant to Resistance. BMC Genomics 20 (1), 245. doi: 10.1186/s12864-019-5625-1 30922224 PMC6438004

[B73] Zhang X. Cao C. Liu Y. Qi H. Zhang W. Hao C.. (2018). Comparative Liver Transcriptome Analysis in Ducklings Infected With Duck Hepatitis A Virus 3 (DHAV-3) at 12 and 48 Hours Post-Infection Through RNA-Seq. Vet. Res. 49 (1), 52. doi: 10.1186/s13567-018-0545-7 29925406 PMC6011267

[B74] Zhang W. Jiang B. Zeng M. Duan Y. Wu Z. Wu Y.. (2020). Binding of Duck Tembusu Virus Nonstructural Protein 2A to Duck STING Disrupts Induction of Its Signal Transduction Cascade To Inhibit Beta Interferon Induction. J. Virol. 94 (9), e01850–e01819. doi: 10.1128/JVI.01850-19 32075929 PMC7163140

[B75] Zhang M. Song K. Li C. Chen Z. Ding C. Liu G. (2015). Molecular Cloning of Peking Duck Toll-Like Receptor 3 (Dutlr3) Gene and its Responses to Reovirus Infection. Virol. J. 12, 207. doi: 10.1186/s12985-015-0434-x 26634454 PMC4668636

[B76] Zhang R. Yang Y. Lan J. Lin S. Xie Z. Zhang X.. (2020a). A Novel Peptide Isolated From a Phage Display Peptide Library Modeling Antigenic Epitope of DHAV-1 and DHAV-3. Vaccines 8 (1), 121. doi: 10.3390/vaccines8010121 PMC7157547 32150877

[B77] Zhang R. Yang Y. Lan J. Xie Z. Zhang X. Jiang S. (2020b). Evidence of Possible Vertical Transmission of Duck Hepatitis A Virus Type 1 in Ducks. Transbound Emerg. Dis. 68 (2), 267–275. doi: 10.1111/tbed.13708 32598568

[B78] Zhao Y. Forst C. V. Sayegh C. E. Wang I. M. Yang X. Zhang B. (2016). Molecular and Genetic Inflammation Networks in Major Human Diseases. Mol. Biosyst. 12 (8), 2318–2341. doi: 10.1039/c6mb00240d 27303926 PMC4955784

[B79] Zhu M. Cai Y. Zhao W. He C. Yang Y. Gao Q.. (2020). Long Noncoding RNAs are Associated With Seneca Valley Virus Infection. Vet. Microbiol. 246, 108728. doi: 10.1016/j.vetmic.2020.108728 32605750

[B80] Zou L. Xu X. Zhai Z. Yang T. Jin J. Xiao F.. (2016). Identification of Downstream Target Genes Regulated by the Nitric Oxide-Soluble Guanylate Cyclase-Cyclic Guanosine Monophosphate Signal Pathway in Pulmonary Hypertension. J. Int. Med. Res. 44 (3), 508–519. doi: 10.1177/0300060516636751 27048385 PMC5536717

